# General Framework for Meta‐Analysis of Haplotype Association Tests

**DOI:** 10.1002/gepi.21959

**Published:** 2016-03-08

**Authors:** Shuai Wang, Jing Hua Zhao, Ping An, Xiuqing Guo, Richard A. Jensen, Jonathan Marten, Jennifer E. Huffman, Karina Meidtner, Heiner Boeing, Archie Campbell, Kenneth M. Rice, Robert A. Scott, Jie Yao, Matthias B. Schulze, Nicholas J. Wareham, Ingrid B. Borecki, Michael A. Province, Jerome I. Rotter, Caroline Hayward, Mark O. Goodarzi, James B. Meigs, Josée Dupuis

**Affiliations:** ^1^ Department of BiostatisticsBoston University School of Public HealthBostonMassachusettsUnited States of America; ^2^ MRC Epidemiology UnitSchool of Clinical MedicineUniversity of CambridgeBox 285 Institute of Metabolic ScienceCambridge Biomedical CampusCambridgeUnited Kingdom; ^3^ Division of Statistical GenomicsDepartment of GeneticsWashington University School of MedicineSt. LouisMissouriUnited States of America; ^4^ Department of PediatricsThe Institute for Translational Genomics and Population SciencesLABioMed at Harbor‐UCLA Medical CenterTorranceCaliforniaUnited States of America; ^5^ Cardiovascular Health Research UnitUniversity of WashingtonSeattleWashingtonUnited States of America; ^6^ Department of MedicineUniversity of WashingtonSeattleWashingtonUnited States of America; ^7^ MRC Human Genetics UnitMRC IGMMUniversity of EdinburghEdinburghUnited Kingdom; ^8^ Department of Molecular EpidemiologyGerman Institute of Human Nutrition Potsdam‐RehbrueckeNuthetalGermany; ^9^ Department of EpidemiologyGerman Institute of Human Nutrition Potsdam‐RehbrueckeNuthetalGermany; ^10^ Generation ScotlandCentre for Genomic and Experimental MedicineInstitute of Genetic and Molecular MedicineWestern General HospitalUniversity of EdinburghEdinburghUnited Kindom; ^11^ Department of BiostatisticsUniversity of WashingtonSeattleWashingtonUnited States of America; ^12^ German Center for Diabetes Research (DZD)Germany; ^13^ Division of EndocrinologyDiabetes and MetabolismCedars‐Sinai Medical CenterLos AngelesCaliforniaUnited States of America; ^14^ General Medicine DivisionMassachusetts General HospitalBostonMassachusettsUnited States of America; ^15^ Department of MedicineHarvard Medical SchoolBostonMassachusettsUnited States of America; ^16^ National HeartLungBlood Institute (NHLBI)Framingham Heart StudyFraminghamMassachusettsUnited States of America

**Keywords:** meta‐analysis, haplotype association tests, family samples, linear mixed effects model

## Abstract

For complex traits, most associated single nucleotide variants (SNV) discovered to date have a small effect, and detection of association is only possible with large sample sizes. Because of patient confidentiality concerns, it is often not possible to pool genetic data from multiple cohorts, and meta‐analysis has emerged as the method of choice to combine results from multiple studies. Many meta‐analysis methods are available for single SNV analyses. As new approaches allow the capture of low frequency and rare genetic variation, it is of interest to jointly consider multiple variants to improve power. However, for the analysis of haplotypes formed by multiple SNVs, meta‐analysis remains a challenge, because different haplotypes may be observed across studies. We propose a two‐stage meta‐analysis approach to combine haplotype analysis results. In the first stage, each cohort estimate haplotype effect sizes in a regression framework, accounting for relatedness among observations if appropriate. For the second stage, we use a multivariate generalized least square meta‐analysis approach to combine haplotype effect estimates from multiple cohorts. Haplotype‐specific association tests and a global test of independence between haplotypes and traits are obtained within our framework. We demonstrate through simulation studies that we control the type‐I error rate, and our approach is more powerful than inverse variance weighted meta‐analysis of single SNV analysis when haplotype effects are present. We replicate a published haplotype association between fasting glucose‐associated locus (G6PC2) and fasting glucose in seven studies from the Cohorts for Heart and Aging Research in Genomic Epidemiology Consortium and we provide more precise haplotype effect estimates.

## Introduction

In recent years, genome‐wide association studies (GWAS) have identified multiple common variants associated with disease and disease‐related traits. In a typical GWAS, association between a trait and genetic variants is tested one variant at a time, and variants with weak association routinely fail to be detected, especially in small cohorts. Therefore, meta‐analysis is often used by large consortia to increase statistical power [Dupuis et al., 2010, Scott et al., 2012, Stram, 1996, Zeggini et al., 2008] to detect variants with a moderate to weak association with the trait of interest. Even with large meta‐analyses, variants identified to date only explain a small proportion of the total heritability. In order to identify the source of the unexplained heritability, emerging approaches have attempted to account for multiple variants at once when evaluating association with a trait. Such approaches include penalized regression methods [Li et al., 2011, Wu et al., 2009], pathway analysis [Holden et al., 2008], gene‐based tests such as burden [Madsen and Browning, 2009] and SKAT [Wu et al., 2010], and haplotype analysis [Liu et al., 2008, Schaid et al., 2002, Tregouet et al., 2004]. The power of these approaches can be enhanced by increasing sample size or combining multiple studies. Methods for meta‐analysis of gene‐based tests are well established and widely used [Hu et al., 2013, Liu et al., 2014], but there are no widely used methods for the meta‐analysis of haplotype association tests.

In this article, we propose a meta‐analysis approach to combine haplotype association results from multiple studies. In the first step of our method, each study provides regression estimates and covariance matrices of haplotype effects, with adjustment for familial correlation to accommodate familial samples or cryptic relatedness. In our second step, cohort‐specific haplotype effect estimates are pooled using a multivariate generalized least square meta‐analysis approach. A global association test and evaluation of the effect of each haplotype can be obtained within our framework. We perform a simulation study to evaluate our approach, comparing results with more traditional meta‐analysis of single‐variant association tests and gene‐based tests. Finally, we replicate a published haplotype association between a fasting glucose‐associated locus (G6PC2) and fasting glucose in seven studies from the Cohorts for Heart and Aging Research in Genomic Epidemiology (CHARGE) Consortium and are able to provide more precise haplotype effect estimates than the prior report involving haplotype estimates from a single cohort [Mahajan et al., 2015]. Code implementing the novel approach, along with a tutorial, is available at http://sites.bu.edu/fhspl/publications/metahaplo.

## Methods

### Haplotype Association Test at Cohort Level

Our approach is based on Zaykin et al.'s [2002] haplotype analysis method for unrelated samples. We incorporate random effects to account for family structure, making the approach applicable to family‐based cohorts, unrelated samples, or a mix of the two. We assume that a total of *n* subjects from a study are sequenced in a region with *q* SNVs and as a result, *K* haplotypes are observed. We assume a general linear (mixed‐effect) model, written as:

(1) $$Y=X\alpha +\beta_{1}h_{1}+...+\beta_{K}h_{K}+b+\varepsilon ,$$

where Y is an n×1 quantitative trait vector, X is an n×p matrix of covariates (without intercept) including, for example, age, sex, and associated genetic principal components controlling for potential population stratification, α is a p×1 coefficient vector for the *p* adjustment variables, each n×1 vector $h_{m}$ (m=1,⋯,K) is the expected haplotype dosage, b is an n×1 random effect vector that accounts for the relatedness within families, and ε is an n×1 vector of the random error terms. When haplotype *m* of the *j*th (j=1,⋯,n) subject is observed, $h_{mj}$, the *j*th entry in $h_{m}$ is either 0, 1, or 2, that is, the number of copies of haplotype *m* the *j*th subject carries. Otherwise, expected haplotype dosages $E[h_{mj}|G_{j}]$ are inferred from $G_{j}$, the q×1 genotype vector of the *j*th subject, using statistical algorithms such as the expectation‐maximization (EM) algorithm [Dempster et al., 1977]. For the *j*th subject, the sum of the *K* haplotype dosages $\sum_{m=1}^{m=K}h_{mj}$ is always equal to 2. The n×1 random effect vector b is assumed to follow a normal distribution $N(0,\sigma_{a}^{2}\Phi )$, where $\sigma_{a}^{2}$ is the additive variance and Φ is the relationship matrix (with entries equal to twice the kinship coefficient for related pairs and 0 for unrelated pairs) derived from pedigree structure or genome‐wide information; in unrelated samples, the matrix Φ reduces to I, the n×n identity matrix. Finally, we assume the vector of error terms ε follows a normal distribution $N(0,\sigma_{e}^{2}I)$, where $\sigma_{e}^{2}$ is the variance of the error term.

Let $X_{o}=\left(X,h_{1},\cdots ,h_{K}\right)$ denote the overall design matrix of size n×(p+K), and define the overall variance matrix as $\Omega =\sigma_{a}^{2}\Phi +\sigma_{e}^{2}I$. The parameters α and $\beta_{k}$ (k=1,⋯,K) are estimated as ${\left(X_{o}^{T}{\hat{\Omega }}^{-1}X_{o}\right)}^{-1}X_{o}^{T}{\hat{\Omega }}^{-1}Y$, where $\hat{\Omega }$ is evaluated at the maximum likelihood estimates $\hat{\sigma_{a}^{2}}$ and $\hat{\sigma_{e}^{2}}$, which can be obtained using the lmekin function in R's coxme package [Therneau, 2012]. The estimated variance of the effect estimates is ${\left(X_{o}^{T}{\hat{\Omega }}^{-1}X_{o}\right)}^{-1}$. The method reduces to an ordinary linear regression when applied to unrelated samples.

### Meta‐Analysis

We assume a total of *N* cohorts participate in the meta‐analysis and the *i*‐th (i=1,⋯,N) cohort provides the estimates $\hat{\beta^{i}}$ and the covariance matrix $\hat{var}\left(\hat{\beta^{i}}\right)$ of the haplotype effects for $K^{i}$ haplotypes, and a total of $K^{\prime }$ haplotypes are observed in at least one cohort. We propose a multivariate meta‐analysis approach [Becker and Wu, 2007] based on generalized weighted least squares to combine the length $K^{i}$ haplotype effect estimates from each cohort, denoted by $\hat{\beta^{i}}$ for studies i=1,⋯,N, into a single effect estimate vector $\tilde{\beta }$ of length $K^{\prime }$. The generalized weighted least square approach is formulated as:

(2) $$\hat{\beta }=\left(\begin{array}{c} \hat{\beta^{1}}\\ \vdots \\ \hat{\beta^{N}} \end{array}\right)=W\beta +e=\left(\begin{array}{ccccc} 1 & ... & 0 & ... & 0\\ 0 & ... & ... & ... & 0\\ 0 & ... & 0 & ... & 1\\ \vdots & \vdots & \vdots & \vdots & \vdots \\ 1 & ... & 0 & ... & 0\\ 0 & ... & 0 & ... & 1 \end{array}\right)\left(\begin{array}{c} \beta^{1}\\ \vdots \\ \beta^{K^{\prime }} \end{array}\right)+e,\;$$

where $\hat{\beta^{i}}$ (i=1,⋯,N) is the $K^{i}\times 1$ haplotype coefficient vector for cohort *i*;

$\hat{\beta }$ is the stacked haplotype coefficient vector from $\hat{\beta^{i}}$ (i=1,⋯,N);

β is the $K^{\prime }\times 1$ coefficient vector of the haplotype effects;

$W=\left(\begin{array}{c} W_{1}\\ \vdots \\ W_{N} \end{array}\right)$ is a $\sum_{i}K^{i}\times K^{\prime }$ design matrix stacked from the *N* cohorts, where ***W***_*i*_ (i=1,⋯,N) is a $K^{i}\times K^{\prime }$ matrix, with zeros and one in each row indicating which haplotype effect is observed by cohort *i*;

e is the error term which is assumed to have a multivariate normal distribution with a mean of **0** and a covariance matrix of $\Sigma =\left(\begin{array}{ccc} var(\hat{\beta^{1}}) & \cdots & 0\\ \vdots & var(\hat{\beta^{k}}) & \vdots \\ 0 & \cdots & var(\hat{\beta^{N}}) \end{array}\right)$.

Note that in the meta‐analysis stage, cohort haplotypes are reordered to match the order assigned to the $K^{\prime }$ haplotypes observed in at least one cohort, and the design matrix W reflects this reordering. Furthermore, because Σ is unknown, in our method, we substitute the sample estimate $\hat{\Sigma }=\left(\begin{array}{ccc} \hat{var}\left(\hat{\beta^{1}}\right) & \cdots & 0\\ \vdots & \hat{var}\left(\hat{\beta^{k}}\right) & \vdots \\ 0 & \cdots & \hat{var}\left(\hat{\beta^{N}}\right) \end{array}\right)$, hence the weighted least square estimator of ***β*** is $\tilde{\beta }={\left(W^{\prime }{\hat{\Sigma }}^{-1}W\right)}^{-1}W^{\prime }{\hat{\Sigma }}^{-1}\hat{\beta }$ and $V=\mathrm{Var}\left(\tilde{\beta }\right)={\left(W^{\prime }{\hat{\Sigma }}^{-1}W\right)}^{-1}$.

### Hypothesis Testing

The global null hypothesis of no association of any haplotype with the trait is expressed as

(3) $$H_{0}:\beta^{1}=\beta^{2}=...=\beta^{K^{\prime }}$$

To construct a test statistic to test for haplotype association, we reparameterize it into the equivalent null hypothesis, where β^1^ is chosen from commonly observed haplotypes:

(4) $$H_{0}:\gamma =\left(\begin{array}{c} \gamma^{1}\\ \vdots \\ \gamma^{K^{\prime }-1} \end{array}\right)=\left(\begin{array}{c} \beta^{2}-\beta^{1}\\ \vdots \\ \beta^{K^{\prime }}-\beta^{1} \end{array}\right)=\left(\begin{array}{c} 0\\ \vdots \\ 0 \end{array}\right)$$

The null hypothesis can be tested using a Wald test statistic of the form

(5) $$\chi^{2}={\hat{\gamma }}^{T}{\left[V^{*}\right]}^{-1}\hat{\gamma },$$

where $\hat{\gamma }$ is estimated from $\tilde{\beta }$ and $V^{*}$ is the covariance matrix of $\hat{\gamma }$, with a dimension of $\left(K^{\prime }-1\right)\times \left(K^{\prime }-1\right)$ and the $jj^{\prime }$th element having the form $V_{jj^{\prime }}^{*}=V_{jj^{\prime }}-V_{j1}-V_{j^{\prime }1}+V_{11}$. Under the null hypothesis, the Wald test statistic follows a $\chi_{{}_{K^{\prime }-1}}^{2}$ distribution asymptotically.

### Cohorts for Heart and Aging Research in Genetic Epidemiology Consortium

The CHARGE consortium comprises multiple studies with the common goal of identifying genes and loci associated with cardiovascular‐related traits. Seven CHARGE cohorts contributed to a meta‐analysis evaluating the association between genetic variants and fasting glucose in 25,305 nondiabetic participants (Table 1). Fasting glucose levels in millimole per liter were analyzed in participants free of type‐2 diabetes. Type‐2 diabetes was defined by cohorts referring to at least one of the following criteria: a physician diagnosis of type‐2 diabetes, on the antidiabetic treatment of type‐2 diabetes, fasting plasma glucose ⩾7 mmol/l, random plasma glucose ⩾11.1 mmol/l, or hemoglobin A1C ≥6.5%. Study‐specific sample exclusions were detailed in [Wessel et al., 2015].

**Table 1:** CHARGE cohorts

**Cohort**	**Sample size**
Generation Scotland: Scottish Family Health Study^a^ (GS)	7,678
Framingham Heart Study^a^ (FHS)	6,561
Cardiovascular Health Study (CHS)	3,525
Family Heart Study^a^ (FamHS)	3,393
Multi‐Ethnic Study of Atherosclerosis (MESA)	2,507
FENLAND (FLD)	1,341
European Prospective Investigation into Cancer and Nutrition, Potsdam (EPIC‐Potsdam)	300
**Total**	25,305

Notes

*^a^*Family‐based cohort.

Genotypes were obtained from the Illumina HumanExome BeadChip [Grove et al., 2013], a genotyping array containing 247,870 variants discovered through exome sequencing in ∼ 12,000 individuals, in which ∼ 75% of the variants are low‐frequency variants (Minor Allele Frequency (MAF) <0.5%). The main content of the chip comprises protein‐altering variants (nonsynonymous coding, splice‐site, and stop gain or loss codons) seen at least three times in a study and in at least two studies providing information to the chip design. We selected four *G6PC2* variants previously studied for their haplotype association with fasting glucose [Mahajan et al., 2015].

### Simulation Studies

To evaluate the validity and power of our approach, we perform a simulation study varying the number of cohorts included in the meta‐analysis (5 or 10), and the type of samples (unrelated, family‐based, mix of the two). We also vary the sample size from 400 up to 1,600 subjects per cohort. See Table 2 for a description of the various study designs investigated in type‐I error rate and power.

**Table 2:** Study designs for type‐I error rate evaluation

**Study design**	**No. of cohort**	**Sample sizes**	Type‐I error rate (G6PC2)	Type‐I error rate (JAZF1)
1	5	250 NF2 (× 5)	0.010	0.010
2	5	250 NFv (× 5)	0.010	0.012
3	5	100 NF2, 175 NF2, 400 U, 700 U, 1000 U	0.013	0.010
4	5	100 NFv, 175 NFv, 400 U, 700 U, 1000 U	0.011	0.011
5	5	100 NFv, 175 NFv, 250 NFv, 325 NFv, 400 NFv	0.011	0.012
6	10	250 NF2 (× 5); 1000 U (× 5)	0.010	0.011
7	10	400 U, 700 U, 1000 U, 1300 U, 1600 U	0.008	0.012
8	5	100 NF2, 175 NF2, 250 NF2, 325 NF2, 400 NF2	0.012	0.011
9	5	250 NF2, 125 NF2 (× 2), 375 NF2 (× 2)	0.011	0.011
		1000 U, 500 U (× 2), 1500 U (× 2)		
10	10	250 NFv (× 7), 1000 U (× 3)	0.012	0.011

Notes

NF2, nuclear family with 2 offspring; NFv, nuclear family with the number of offspring randomly selected to be between 1 and 4; U, unrelated subjects.

Simulated trait values are dependent on sex, age, and haplotypes/genetic variants (power evaluation only). Sex of mothers and fathers (founders) are fixed in a heterosexual marriage but are randomly assigned to offspring, with equal probability. The age for unrelated individuals and the first offspring in a family are generated from a uniform distribution over the range 30 to 50. Additional offspring's ages are set to be within 5 years of the first offspring with at least a 1 year gap (no twins), using a uniform distribution. For family samples, the age of the mother is restricted to be 20–45 years older than her offspring, and the father's age to be within 5 years of the mother's age, with a restriction that the age be at least 20 years older than the older offspring.

We select the known T2D‐associated genes *G6PC2* (chromosome 2; Tables 3 and 4) and *JAZF1* (chromosome 7; Tables 5 and 6) to generate the reference panel haplotypes (Tables 3 and 4). We use the observed haplotypes and frequencies estimated by EM algorithm from 6561 participants from the Framingham Heart Study. For example, in *JAZF1* no single haplotype has a frequency greater than 25% and eight haplotypes have frequency greater than 1% (Table 6).

**Table 3:** *G6PC2* variants

Name	Chr	MapInfo	dbSNPID	Minor	Major	FHS MAF
exm‐rs560887	2	169763148	rs560887	A	G	0.293
exm239664	2	169763262	rs138726309	T	C	0.0036
exm239667	2	169764141	rs2232323	C	A	0.0078
exm239672	2	169764176	rs492594	C	G	0.4553

**Table 4:** *G6PC2* haplotype frequencies

rs560887	rs138726309	rs2232323	rs492594	FHS frequency
C	C	A	C	0.46
T	C	A	G	0.29
C	C	A	G	0.24
T	C	C	G	0.006
C	T	A	C	<0.001
T	C	A	C	<0.001
C	T	A	G	<0.001
C	C	C	G	<0.001

**Table 5:** *JAZF1* variants (chromosome 7)

Name	Position	dbSNPID	Minor	Major	MAF
exm‐rs10486567	27976563	rs10486567	A	G	0.2415
exm2270592	28039797	rs38523	C	T	0.3683
exm‐rs864745	28180556	rs864745	G	A	0.4965
exm‐rs1635852	28189411	rs1635852	C	T	0.4973
exm‐rs849134	28196222	rs849134	G	A	0.4917

**Table 6:** *JAZF1* haplotype frequencies

Haplotype	rs10486567	rs38523	rs864745	rs1635852	rs849134	Frequency
1	G	T	A	T	A	0.2327
2	G	T	G	C	G	0.2295
3	G	C	G	C	G	0.1608
4	G	C	A	T	A	0.1295
5	A	T	A	T	A	0.0866
6	A	T	G	C	G	0.0793
7	A	C	A	T	A	0.0434
8	A	C	G	C	G	0.0259
9	A	T	G	T	A	0.0029
10	A	T	A	C	A	0.0029
11	A	C	A	C	A	0.0023
12	G	T	A	C	A	0.0019
13	G	T	G	T	A	0.0017
14	G	C	G	T	A	0.0005

Genotypes are simulated by randomly assigning a pair of haplotypes to founders, and by dropping randomly selected haplotypes to offspring assuming no recombination within haplotypes. Although phasing information is available in our simulation setting, we do not use the phase information when implementing our approach because such information is not typically available in real datasets. We use the EM algorithm to infer expected haplotype dosage conditional on genotypes via R package haplo.stats [Sinnwell and Schaid, 2013].

When estimating haplotype effects at the cohort‐level, rare haplotypes (frequency<0.1%) are collapsed to stabilize the computation and to avoid potential singularities due to high LD among SNVs.

#### Type‐I Error Rate

For evaluating the type‐I error rate of our new approach, a trait unassociated with the haplotypes is simulated using a multivariate normal distribution with mean $\hat{\mu }=0.02\times \mathrm{age}+0.5\times \mathrm{sex}$ (sex is set to 1 for males and to 2 for females) and a covariance matrix $\sigma_{a}^{2}\Phi +\sigma_{e}^{2}I$, with $\sigma_{a}^{2}=\sigma_{e}^{2}=0.5$. Age and sex explained about 10% and 5% of the trait variance, respectively, resulting in a trait with moderate heritability ($h^{2}=\frac{\sigma_{a}^{2}}{Var(Y)}\approx 0.42$).

Cohort‐specific analyses are performed by first estimating haplotypes using the EM algorithm implemented in the R package haplo.stats, followed by regression analysis using haplotype dosages and covariates as independent variables. Cohort results are then meta‐analyzed using the novel approach previously described, and the global association test *P* values are recorded. Ten thousand simulations are performed to assess the type‐I error rate in all scenarios at the nominal threshold α=0.01 (Table 2).

#### Power Evaluation

The power of our novel haplotype meta‐analysis approach is evaluated in a total of 16 scenarios (phenotype datasets) divided into four study designs (study design 1–4 from Table 2), with varying haplotype or SNV effects. For each scenario, we first compute the meta‐analysis haplotype global test statistic, and then compare to meta‐analysis of both single variant tests and gene‐based tests. For single variant tests, we compute the meta‐analysis test statistic using inverse‐variance weighted method that has been shown to be the most powerful when the effect size is constant across cohorts [Zhou et al., 2011]. We then select the SNP with the minimum meta‐analysis *P*‐value $P_{min}=min_{\left\{1\leq i\leq K\right\}}P_{i}$ (K=4 for *G6PC2*; K=5 for *JAZF1*) and adjust the meta‐analysis *P*‐value for multiple testing using a Bonferroni correction for the effective number of independent variants [Gao et al., 2008]. We denote the result for the best SNP in the single variant analysis by “min *P*”. For gene‐based tests, we choose SKAT and Burden test with Wu weights and perform the analysis using R package seqMeta [Voorman et al., 2014]. We use α=0.001 to evaluate the power of all four approaches.

For each scenario, the phenotype is simulated using a multivariate normal distribution with mean $\hat{\mu }$ and a covariance matrix $\sigma_{a}^{2}\Phi +\sigma_{e}^{2}I$, with $\sigma_{a}^{2}=\sigma_{e}^{2}=0.5$, but unlike the type‐I error scenarios, the value of $\hat{\mu }$ depends on genotypes/haplotypes in addition to the covariates of age and sex. We investigate four genetic effect scenarios: one or two causal genetic variants, or one or two causal haplotypes. For the causal variant scenario, $\hat{\mu }=0.02\times \mathrm{age}+0.5\times \mathrm{sex}+b_{g_{1}}\times g_{1}+b_{g_{2}}\times g_{2}$ where **g_j_** (j=1,2) is a vector containing the number of minor alleles (0, 1, or 2) carried by individuals in the sample, and $b_{g_{j}}$ is the effect of variant *j*, set to $\sqrt{\frac{R^{2}}{4MAF_{j}\left(1-MAF_{j}\right)}}$, where $MAF_{j}$ is the minor allele frequency of variant *j* and $R^{2}=0.01$ is the proportion of variance explained by this specific variant (haplotype). When only one causal variant is included in the model, $b_{g2}=0$ and $b_{g1}$ is multiplied by $\sqrt{2}$. For the causal haplotype models, $\hat{\mu }=0.02\times \mathrm{age}+0.5\times \mathrm{sex}+b_{h1}\times h_{1}+b_{h2}\times h_{2}$, where $h_{j}$ is a vector containing the number (conditional dosage) of haplotype *j* carried by individuals in the sample, and $b_{hj}$ is the effect of haplotype *j*, set to $\sqrt{\frac{R^{2}}{2{\bar{h}}_{j}\left(1-{\bar{h}}_{j}/2\right)}}$, where ${\bar{h}}_{j}$ is mean haplotype dosage of haplotype *j* and $R^{2}=0.01$. When only one causal haplotype is included in the model, $b_{h2}=0$ and $b_{h1}$ is multiplied by $\sqrt{2}$. For the *JAZF1* gene, we select two haplotypes, GTATA (the most frequent haplotype) and GCGCG (the third most frequent haplotype), to have an effect on the phenotype while all other haplotypes have no effect on the phenotype. For models with single variant effects, we select rs849134 and rs38523 to have nonzero effect on the trait, while all other genetic variants have no effect. For the *G6PC2* gene, we select CCAC and TCAG, the two most frequent haplotypes to have an effect on the phenotype. For models with single variant effects, we select rs560887 and rs2232323 to have nonzero effect on the trait.

A thousand simulations with five independent cohorts are performed to compare the power of our approach to the single variant method adjusted for multiple testing and gene‐based methods.

## Results

### Meta‐Analysis of Four Coding Variants on G6PC2 Region

*G6PC2* is a known locus to affect fasting glucose level. Among the 17 exonic variants on the exome chip, 15 are rare variants (MAF<1%) and two are common variants (rs560887 with MAF = 25.4%; rs492594 with MAF = 43.7%). Previous GWAS have identified the A allele of rs560887, one of the two common variants to be associated with lower fasting glucose level ([Bouatia‐Naji et al., 2008]: β=−0.07 mmol/l, $p=6\times 10^{-16}$; [Dupuis et al., 2010]: β=0.075±0.003 mmol/l, $p=8.5\times 10^{-122}$). A recent large‐scale exome‐chip analysis indicated that these 15 rare variants also had a joint effect on fasting glucose [Wessel et al., 2015]. Our approach is applied to study the association between the haplotype structure of four coding variants rs560887, rs138726309, rs2232323, and rs492594 and fasting glucose, using CHARGE exome‐chip data. We perform a meta‐analysis of seven studies comprising of three family‐based and four population‐based cohorts with up to 25,305 non‐diabetic European participants, to better understand how the overall haplotype structure as well as how the single haplotype affect fasting glucose level. With a meta‐analysis sample size of 25,305, we have successfully replicated a previous reported haplotype analysis of four coding variants on G6PC2 region [Mahajan et al., 2015], but with higher precision (Table 7). Our effect size estimates are consistent with previously published estimates, in terms of both direction and magnitude. However, prior results were based on a single population‐based cohort with 4,442 participants. In contrast, our analysis is based on seven cohorts with over 25,000 participants. Among the five haplotypes shared by all seven studies, one copy of the most significant haplotype, TCAG, decreases fasting glucose levels by 0.074 (95% confidence interval (CI): 0.063,0.085) mmol/l, on average; one copy of the second most significant haplotype, CCAG, increases the average fasting glucose levels by 0.039 (95% CI: 0.028,0.050) mmol/l; and one copy of the third most significant haplotype, TCCG, decreases fasting glucose levels by an average of 0.12 (95% CI: 0.065,0.18) mmol/l. Most haplotype effect estimates reported in Mahajan et al. [2015] fall within our 95 % CI, with the exception of estimates for TCCG (Mahajan et al.'s [2015] estimates = 0.205), which fall just outside our reported CI.

**Table 7:** Single haplotype association test using 4SNVs on G6PC2 region

rs560887	rs138726309	rs2232323	rs492594	β (SE)	*P*‐value	Frequency	$\beta_{M}$($SE_{M}$)^a^
C	C	A	C			0.4394	
T	C	A	G	−0.073 (0.0055)	$4.56\times 10^{-41}$	0.2671	−0.065(0.011)
C	C	A	G	0.039 (0.0056)	$5.98\times 10^{-12}$	0.2645	0.034(0.012)
T	C	C	G	−0.12 (0.029)	$2.82\times 10^{-5}$	0.0065	−0.205(0.057)
C	T	A	C	−0.022 (0.056)	0.70	0.0021	−0.202(0.077)
T	C	A	C	−0.031 (0.020)	0.12	0.0195	NA

Notes

The haplotypes are observed in all cohorts except that the last one is observed only in FHS, CHS, GS, and FamHS.

*^a^* $\beta_{M}$ and $SE_{M}$ denote the estimates from the paper of Mahajan et al. [2015].

### Simulations

Ten scenarios with increasing diversity in the study designs of the cohorts included in the meta‐analysis are simulated to evaluate type‐I error rate of our approach. The type‐1 error rate is well controlled in all scenarios investigated (Table 2).

**Figure 1:**
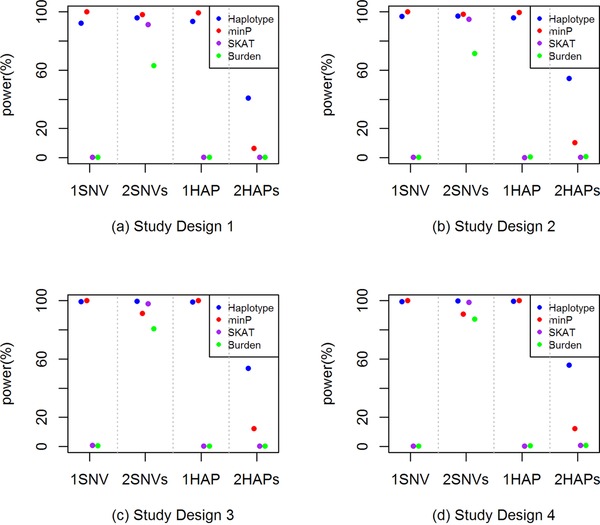
Power of the haplotype meta‐analysis approach compared to gene‐based methods and single SNV meta‐analysis (min *P*) adjusted for multiple testing in the *G6PC2* region, evaluated at α=0.001 in four study designs. Description of the four study designs used in the simulation can be found in Table 2 (study design 1–4). The labels on the *x* axes denote that 1 (SNV) or 2 (2SNVs) SNVs are influencing the phenotypes, or 1 (1HAP) or 2 (2HAPs) haplotypes have an effect on the phenotypes.

**Figure 2:**
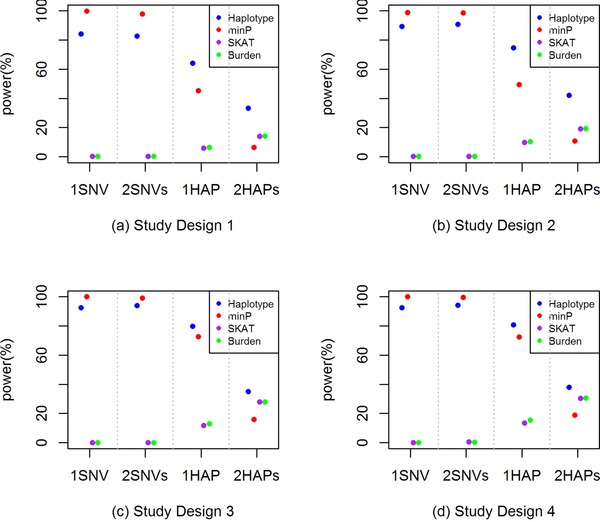
Power of the haplotype meta‐analysis approach compared to gene‐based methods and single SNV meta‐analysis (min *P*) adjusted for multiple testing in the *JAZF1* region, evaluated at α=0.001 in four study designs. Description of the four study designs used in the simulation can be found in Table 2 (study design 1–4). The labels on the *x* axes denote that 1 (SNV) or 2 (2SNVs) SNVs are influencing the phenotypes, or 1 (1HAP) or 2 (2HAPs) haplotypes have an effect on the phenotypes.

In the simulations to evaluate power, our approach is shown to be almost as powerful as the single SNV approach when SNVs are influencing the trait, but much more powerful to detect true haplotype effects. For example, in the family based design scenarios, our approach is 40% more powerful than single SNV analyses when two haplotypes have nonzero effect on the phenotypes (Figures 1 and 2). A similar pattern is observed for designs with a mix of unrelated and related samples. The gain in power is smaller when a single haplotype is influencing the trait, but present for all scenarios evaluated. When compared to the gene‐based tests, our approach is uniformly more powerful in all scenarios across all study designs (Figures 1 and 2) because of the Wu (default) weighing scheme that downweights common variants.

## Discussion

We have proposed a general meta‐analysis approach to combine the haplotype association results from multiple cohorts. Our approach imposes no restrictions on the haplotypes observed across cohorts. Instead, our approach can incorporate information from haplotypes observed in a single cohort in addition to haplotypes observed in multiple cohorts. In the first stage of our approach, haplotype association analysis is performed at the cohort level. Information about the haplotype structure, frequencies, effect estimates, and covariance of effect estimates is collected, and meta‐analyzed in the second stage using a generalized weighted least square approach. The association between a trait and any single or multiple haplotypes can be easily evaluated within our framework.

We evaluated the type‐I error rate in a variety of scenarios with different cohort designs that included a mix of unrelated and family samples. Type‐I error rate was controlled in all scenarios investigated. We also compared the power of our approach with single variant tests corrected for multiple testing (min P approach), and demonstrated that our approach had equivalent power when variants, not haplotypes, influenced the trait, but was more powerful in the presence of true haplotype effects. Our haplotype approach also provided more evidence for association compared to gene‐based tests applied with the default weighting scheme, as exemplified in a recent large‐scale exome‐chip project [Wessel et al., 2015] applied to the G6PC2 region comprising 15 rare variants (MAF<1%). Our simulations also illustrated that the haplotype effect size estimates obtained from meta‐analysis were unbiased, even when family‐based cohorts were included.

While our approach cannot serve as the only tool for the discovery of associated variants and regions, it is a complementary tool to single‐variant and gene‐based tests. Mahajan et al. [2015] demonstrated the usefulness of haplotype analysis in their investigation of the effect of *G6PC2* variants on fasting glucose. In 4,442 nondiabetic subjects from the Oxford Biobank, the G allele from the coding variant rs492594 appears to significantly decrease fasting glucose levels. However, when conditioning on the variant with the largest effect (rs560887) on fasting glucose, the effect estimates of the G‐allele from rs492594 is reversed, and the G allele appears to decrease fasting glucose, an apparent paradox. However, looking at the haplotype estimates elucidates the mystery: the rs492594 G allele is most frequently observed on the same haplotype as the glucose raising allele (T) from the strongest associated variant (rs560887), giving the impression that the G allele also increases fasting glucose. Our analysis supports this conclusion, and refines the effect estimates provided by Mahajan et al. [2015] by increasing the number of samples used to obtain effect estimates via meta‐analysis, providing more precise estimates, as reflected in the smaller standard errors.

Our approach has some limitations. The variants included in the haplotype analysis must be genotyped or imputed in all cohorts. In other words, all cohorts must include the same set of variants in their analysis. Moreover, when using imputed genotypes, best‐guess genotypes must be used because the approach does not currently handle genotypes in the form of dosage. The EM algorithm currently employed for inferring haplotypes works best for a moderate number of variants (< 15), and very rare haplotypes (frequency<0.1%) are recommended to be collapsed to ensure computation stability. Despite these limitations, our approach has the potential to shed some light on the relationship between traits and multiple associated SNVs in a region.
